# Attitudes and Feelings towards the Work of Teachers Who Had a School Nurse in Their Educational Center during the COVID-19 Pandemic

**DOI:** 10.3390/ijerph20043571

**Published:** 2023-02-17

**Authors:** Borja Nicolás Santana-López, María Desamparados Bernat-Adell, Luciano Santana-Cabrera, Esteban Gabriel Santana-Cabrera, Gloria Raquel Ruiz-Rodríguez, Yeray Gabriel Santana-Padilla

**Affiliations:** 1 Intensive Care Unit, Hospital Universitario de Gran Canaria Doctor Negrín, 35010 Las Palmas de Gran Canaria, Spain; 2 Nursing Department, Universitat Jaume I, 12006 Castellón de la Plana, Spain; 3 Intensive Care Unit, Complejo Hospitalario Universitario Insular Materno-Infantil de Gran Canaria, 35016 Las Palmas de Gran Canaria, Spain; 4 Colegio de Educación Infantil y Primaria Los Giles, 35010 Las Palmas de Gran Canaria, Spain; 5 Consejería de Educación, Universidades, Cultura y Deportes, 35001 Las Palmas de Gran Canaria, Spain; 6 Emergency Surgery Unit, Complejo Hospitalario Universitario Insular Materno-Infantil de Gran Canaria, 35016 Las Palmas de Gran Canaria, Spain

**Keywords:** attitudes, COVID-19, educational personnel, emotions, pandemics, nurses, community health, nurses, public health, work engagement, work performance

## Abstract

The objective of this study was to analyze the influence of the availability of a health professional on the beliefs, attitudes, and work feelings of teaching staff when facing the COVID-19 pandemic. This is a two-phase study: In the first one, the Delphi technique was used to update an instrument used by the authors in a previous investigation in 2020. The second phase was a cross-sectional, descriptive, and comparative study, carried out through an electronic questionnaire distributed among the teaching staff of the Autonomous Community of the Canary Islands (Spain), during the first two months of the 2021/22 academic year, in the midst of the fifth wave of COVID-19. Data were analyzed using Pearson’s chi-squared test and the linear trend test. The reasons for advantages were analyzed and the dimensions of the questionnaire were compared between the groups studied (with or without a healthcare professional in the center). Out of 640 teachers in the study, 14.7% (*n* = 94) stated that they had a reference professional with health training in their educational center (a school nurse) for the management of possible cases of COVID-19. Significant differences were found in five of the nine dimensions studied between the groups of teachers analyzed. Teachers who had a health professional, specifically a nurse, during the pandemic indicated that they felt safer in their educational center, as they perceived that they had more personal protective equipment (OR = 2.03, [95% CI: 1.23–3.35]; *p* = 0.006). They were also more committed (OR = 1.89, [95% CI: 1.04–3.46]; *p* = 0.038) with their educational work and assumed more obligations (OR = 1.87, [95% CI: 1.01–3.44]; *p* = 0.045) and risks (OR = 2.82, [95% CI: 1.13–7.07]; *p* = 0.027). In addition, they presented fewer feelings of burnout (OR = 0.63, [95% CI: 0.41–0.98]; *p* = 0.041). These results indicate that having nurses in educational centers improves teachers’ ability to cope with a pandemic situation.

## 1. Introduction

The educational field has been adapting to the measures decreed by the health authorities since the COVID-19 pandemic began, to safeguard the health of teachers and students. These changes have meant a modification in teachers’ work routines, assuming new functions that, if it were not for the pandemic, they would not have had to perform.

The declaration of the state of alarm by the Spanish Government, in order to reduce the spread of infections, on March 14, 2020, meant that around 840,000 teachers replaced face-to-face teaching with distance teaching, making use of information and communication technologies (ICT), in the final stretch of the 2019/20 academic year, so as not to hinder the educational process [1,2].

According to Solís-García et al., the fact that teachers had to teach online caused an imbalance between their work and family life, with women being the ones who perceived to a greater extent that this way of working had a negative impact on their life. In addition, the authors pointed out that it was the teachers from public institutions who perceived less organizational support, who reported higher levels of anxiety related to techno-stress, and who had greater negative interactions between family and work [3].

In a study carried out in May 2020, prior to the start of the 2020/21 academic year, the same authors determined that teachers in the Canary Islands were willing to work face to face, as long as they were guaranteed hygiene and safety measures in their workplace. However, many of them did not believe that they would have these measures available when the course began [4]. Teachers, despite knowing the measures to prevent contagion, had a certain lack of knowledge in terms of hygiene and infection prevention [4].

On 15 September 2020, students and teachers returned to the classrooms after the confinement period, under different health and safety guidelines (masks, ventilation, safety distance, reduced ratios in the classrooms, bubble groups, etc.) given to teachers through the publication of a model contingency plan against COVID-19, prepared by the Ministry of Education, Universities, Culture and Sports of the Autonomous Government and which was intended to be a guide to facilitate teaching during the pandemic in educational centers [5,6].

During this return to atypical face-to-face education, teachers had to assume, in addition to their usual teaching activity (occasionally alternating it with telematic teaching), additional tasks, such as the implementation of hygienic-sanitary and safety measures and even assume the management of possible positive cases of COVID-19; these demands are considered as additional stressors and could cause anxiety for teachers, according to Pressley et al. [7]. A study carried out in the Basque Country (Spain), with a sample of 1633 teachers from all educational fields, determined that half of them suffered stress and anxiety and more than 30% of them suffered from depression during the first month of the academic year 2020/21 [8].

The fact that teachers took on tasks other than teaching is probably related to the lack of a healthcare professional, such as a school nurse, in Spanish classrooms. Spain has one school nurse for every 8497 students, and specifically, in the Autonomous Community of the Canary Islands, 10 nurses for every 326,105 students, according to data from the School Nursing Observatory of the General Nursing Council (Consejo General de Enfermería, CGE) [9].

On the contrary, this health professional is fully standardized, regulated, and implemented in other European countries, such as the United Kingdom and Finland, which establish a ratio of one school nurse for every 1155 or 600 students, respectively [10]. Likewise, the National Association of School Nurses recommends the following ratios: 1:750 for the general population of a school, 1:225 in students with complex health needs and who require care, and 1:125 in students with highly complex health problems [11].

School nurses, before the COVID-19 pandemic, worked towards promoting a healthy and safe environment in their educational centers by providing health education, interventions for acute and chronic illnesses, prevention of diseases related to lifestyles and sexually transmitted diseases, case management, and other public health activities between the students, their parents, and the educational staff [12]. In addition, these nursing interventions are associated with positive changes in student’s health and in their academic performance in multiples studies carried out in the United States of America [12].

On the other hand, Martinsson et al. described in two studies that the school nurses in Sweden played an important role during the current pandemic by listening and providing reliable health information to students and teachers who were worried about contracting or transmitting the disease to their families, alleviating their concerns [13]. In addition, school nurses cooperated with teachers to implement the pertinent hygiene, health, and safety measures stipulated by the Public Health Agency to reduce infections in schools [14]. They also had online health consults with those students confined to their homes through video calls or by telephone, monitoring the evolution of their infection [14].

Several studies show that there are numerous activities and interventions carried out by school nurses in different countries (before and during the pandemic) and how they have a positive impact on students and teachers [12,13,14]. However, experts in the field of school nursing suggest that there are still not enough studies that quantify the impact of the school nurse in educational centers [15].

The originality of this research lies in the fact that, in Spain, to date, there are no studies on whether the presence of a health professional (a school nurse) in educational centers implies any benefit for teaching staff and even more so during a socio-sanitary crisis, such as the COVID-19 pandemic. In accordance with the above, the objective of the present study is to analyze the influence of this health professional on the beliefs, attitudes, and work feelings of the Canary Islands’ teaching staff during the pandemic.

## 2. Materials and Methods

This study used a mixed methodology with two differentiated phases.

### 2.1. Phase One

In the first phase, the Delphi technique was used to update the instrument used by the authors in a previous investigation in 2020, during the deconfinement process of the first wave [4].

After adapting it to the current circumstances, a first version of the questionnaire was generated that sought to know the beliefs, attitudes, and feelings of the teaching staff towards work after five waves of the COVID-19 pandemic. This first questionnaire was distributed to a group of teaching experts (*n* = 14), belonging to different educational fields, who, after two rounds, agreed on the final instrument, evaluating its internal validity. The adapted questionnaire was composed of 69 items divided into five blocks of questions.

The first block was on the beliefs of teaching staff about the pandemic, the second on attitudes regarding the work performed, a third on sociodemographic questions, a fourth block on work issues of the interviewee (among which was whether their workplace had a reference professional with health training for the management of possible COVID-19 cases), and finally, the fifth block on feelings towards work. To determine the reliability of the scale, the Cronbach’s alpha coefficient of the 69 items was assessed and it was 0.967.

When the study period began, in week 38/2021, the cumulative incidence (CI) at the national level was 24.6 (11,662 cases per 100,000 inhabitants) and in the Canary Islands, it was 22.5 (503 cases per each 100,000 inhabitants) [16]. Likewise, CI decreased until the end of the study period during week 42/2021 to 20.7 (9816 cases per 100,000 inhabitants) and 21.7 (485 cases per 100,000 inhabitants) in Spain and the Canary Islands, respectively [17]. Therefore, the work was developed during the fifth epidemiological period of the COVID-19 pandemic in Spain.

### 2.2. Phase Two

During the second phase, a quantitative methodological approach was applied, carrying out a descriptive cross-sectional study, using an electronic questionnaire, in the region of the Autonomous Community of the Canary Islands, Spain. The study period lasted from 24 September to 25 October 2021, during the fifth wave of the COVID-19 pandemic in this region.

The questionnaire was digitized in “*Google Form*” format and was pre-tested by the members of the research team to detect misunderstandings. As it was a self-administered instrument, a brief explanation of the study was included in the first part, requesting express consent to participate in the research, also informing the participants that it was an anonymous questionnaire and thanking them for their collaboration.

It was distributed to the teaching staff of the Canary Islands by telematic means. In order to reach the target population, reference workers were contacted in primary education centers, secondary education institutes, and universities, who collaborated in the dissemination.

The target population was composed of teachers from any public or private educational institution, from primary and preschool teachers to university professors. The sample size was calculated considering a population of 35,714 teachers who were working at the time of carrying out the study in the Autonomous Community [18,19]. A minimum sample size of 469 teachers was calculated, with a sample error of 4.5% and a confidence index of 95%.

A total of 665 completed questionnaires were received, of which 25 were excluded because the participants did not give their consent to include their answers in the study or because they responded inadequately to a control question (designed to avoid automatic answers), obtaining a final sample of 640 teachers, implying a sampling error of 3.84% for a confidence index of 95%.

### 2.3. Data Processing and Statistical Analyses

Demographic and employment characteristics were described by the absolute and relative frequencies of their component categories when those characteristics composed nominal variables; and with a median (P_25_–P_75_) when they were scale variables that were not normally distributed (verified with the Kolmogorov–Smirnov test).

The global responses given to the items on the form were described with the general absolute and valid relative frequencies. After performing the global descriptive analysis of the sample, the participants were assigned to two groups: those teachers who stated that they had a health professional in their educational center (school nurse) (*n* = 94) and those who did not have one (*n* = 546).

Differences in demographic and employment characteristics between the two groups were compared using Pearson’s chi-squared test or Fisher’s exact test, where appropriate, for characteristics in the form of nominal variables, and using the Mann–Whitney U test when its form was non-normal scale variables.

The differences in the responses given to the items in the Beliefs, Attitudes, and Feelings sections between teachers with and without a school nurse in their educational center were compared with Pearson’s chi-squared test or Fischer’s exact test, as appropriate. If statistical significance was reached, the differences in responses for all demographic and employment variables were compared using Pearson’s chi-squared test, Fisher’s exact test, or the Mann–Whitney U test according to the shape and distribution of those variables.

In the case of reaching statistical significance, the odds ratios were obtained with their confidence intervals at 95% of the response given to the item by the teacher who had this health professional in their center, with respect to those teachers who did not have this health professional, by adjusting multivariable binary logistic regression models, with the response as an effect or dependent variable, and the type of teacher (with or without a nurse) as an explanatory variable for the response, adjusted for all demographic and employment characteristics, which reached statistical significance between each type of teacher. These models employed the step-back strategy with full baseline models and the Wald covariate input–output criterion.

Finally, the technique of discriminant exploratory factor analysis of principal components was used to extract possible latent dimensions in the set of items from the Beliefs, Attitudes, and Feelings sections. The adequacy of the use of this technique was verified by adjusting the data to Bartlet’s sphericity and the Kaiser–Meyer–Olkin (KMO) sample adequacy test.

For the detected components, the relative frequency of variance of the accumulated responses and the composition of factorial loads by varimax rotation on the identified dimensions are presented. Responses with a negative load factor were recoded, inverting the direction of the response so that they all carried the same direction. With the items that made up the dimensions, dimensions were created by quantitative agglomeration in the form of numerical scales and a global evaluation of the form by composition of the dimensions (See Appendix A, Table A1).

The data fit test for an exploratory principal component factor analysis, firstly, for the Beliefs section, showing a KMO agreement measure of 0.645 and a Bartlet sphericity of 259 (*p* < 0.001) for three dimensions that explain 61% of the variance of the responses. These dimensions were Coping Capacity, Prevention, and Organizational Changes.

For the Attitudes section, the KMO measure of agreement was 0.720 with a Bartlet sphericity of 636 (*p* < 0.001) for three dimensions that explain 46% of the variance of the responses. These dimensions were Risks, Commitments, and Obligations.

For the Work Feelings section, a KMO of 0.799 and a Bartlet sphericity of 1979 (*p* < 0.001) were obtained for three dimensions that explain 48% of the variance of the responses. These dimensions were Burnout, Supports, and Job Satisfaction.

The dimensions of each section and its global output scale in numerical format were compared between the two groups of teachers. After obtaining statistical significance in these exploratory comparisons, the dimensions and global scale of the forms were converted into dichotomous qualitative variables using a cut-off point of 75% of their numerical value. For the dimensions and the global output of each section, the same procedure carried out with the items was repeated, obtaining the significance of the differences in dimensions and global output of each section between teachers with and without a nurse in the educational center and their odds ratios in 95% confidence intervals, adjusted by logistic regression.

All of the contrast tests applied in these data processes were bilateral at a level of statistical significance *p* ≤ 0.05, except for the decision to include a demographic or employment characteristic as a covariate of adjustment in the logistic regression models, which was *p* ≤ 0.10, to rule out any possible interactions between these factors.

All calculations were performed using the IBM^®^ SPSS 25.0^TM^ (IBM Corp, Armonk, NY, USA) statistical software.

## 3. Results

### 3.1. Sample Profile

The majority were women (*n* = 477; 74.5%), with a mean age of 47.8 ± 9.0 years, who lived with their partner and children (*n* = 293; 45.8%). Most had permanent contracts (*n* = 433; 67.6%) and a mean work experience as teachers of 18.45 ± 9.9 years. Almost all participants had been vaccinated against COVID-19 (*n* = 622; 97.2%), 29.7% had been isolated at home after being in contact with positive people or due to compatible symptoms (*n* = 190), and 4.4% (*n* = 28) contracted the disease. A total of 11.1% (*n* = 71) required psychological or psychiatric support at some point during the pandemic. A total of 38.1% (*n* = 244) lived with vulnerable people or with risk pathologies, and 25.3% (*n* = 162) were people at risk.

When both groups of teachers, with (*n* = 94; 14.7%) or without (*n* = 546; 85.3%) a school nurse at the center, were compared, no statistically significant differences were found for most of the sociodemographic and work variables analyzed (See Appendix A, Table A2). However, the teachers who had this healthcare figure in their center lived in a lower proportion with vulnerable people or with pathologies considered to be at risk of COVID-19 infection (27.7% vs. 39.9%; *p* = *0.029*), and themselves were people at lower risk of the disease (14.9% vs. 27.1%; *p* = *0.012*). (See Appendix A, Table A3).

### 3.2. Differences Found in the Items from the Beliefs, Attitudes, and Feelings towards Their Work

In general, 58.0% (*n* = 347) believed that they had adequate personal protective equipment (PPE) that protected them from contracting the disease. It should be noted that this percentage was significantly higher among the group of teachers who had a school nurse in their center (71.6% vs. 55.7%; *p* = 0.005), (OR = 2.03, [95% CI: 1.23–3.35]; *p* = 0.006).

As far as the education system is concerned, despite the fact that half of the teachers considered that the system had adequately coped with the pandemic (*n* = 320; 56.0%), the group who had this healthcare professional in their center gave a significantly more positive assessment of how the education system had dealt with the pandemic (66.7% vs. 54.1%; *p* = 0.030), (OR = 2.00, [95% CI: 1.28–3.12]; *p* = 0.030).

Regarding the salary they received, the teachers who had a school nurse in their center were significantly more satisfied with their salary (57.4% vs. 39.7%; *p* = 0.004), (OR = 1.95, [95% CI: 1.24–3.04]; *p* = 0.004). (See Appendix A, Table A4).

A total of 71.3% (*n* = 342) of the teaching staff were not willing to work if there was a higher than usual risk of becoming infected at work and contracting COVID-19. In addition, 82.9% (*n* = 395) were not willing to work if the risk of infecting their relatives increased. However, the teachers who had a health figure in their center stated, in a greater proportion, that they would probably go to work despite the fact that there was a higher than usual risk of infecting themselves (38.4% vs. 27.0%; *p* = 0.049) (OR = 1.68, [95% CI: 1.01–2.83]; *p* = 0.049), or their relatives (27.8% vs. 15.3%; *p* = 0.009) (OR = 2.12, [95% CI: 1.19–3.81]; *p* = 0.010).

A higher proportion of teachers who had this health professional in their center agreed with the statement that the educational staff had the duty to continue working despite the risk (43.6% vs. 30.8%; *p* = 0.014), (OR = 1.74, [95% CI: 1.11–2.72]; *p* = 0.015), and that those who refuse to work during the pandemic should somehow be sanctioned (43.6% vs. 29.5%; *p* = 0.006), (OR = 1.85, [95% CI: 1.18–2.89]; *p* = 0.007).

Only a little more than half of the teachers noted that their company provided them with the necessary material means to perform their work (*n* = 360; 56.3%). This proportion rose among those who had a nurse in their center, (68.1% vs. 54.2%; *p* = 0.008), (OR = 1.80, [95% CI: 1.13–2.87]; *p* = 0.013) (See Appendix A, Table A4).

Finally, no significant differences were found between the compared groups in the items from the Work Feelings section.

### 3.3. Differences between Dimensions

When the Beliefs, Attitudes, and Feelings were analyzed, grouped into dimensions, significant differences were found in five of the nine dimensions.

For the Coping Ability dimension in the Beliefs section, 42.6% of positive responses were obtained among teachers who had a school nurse at the center, compared to 29.2% among those who did not have one (*p* = 0.027), for an adjusted OR of positive response of 1.81 (95% CI: 1.07–3.06; *p* = 0.028) in favor of teachers with a health figure.

For the Risk dimension, within the Attitudes section, 33.3% of positive responses were obtained among teachers who had a school nurse at their center, compared to 15.0% among those who did not have one (*p* = 0.023), for an Adjusted OR of positive response of 2.82 (95% CI: 1.13–7.07; *p* = 0.027) in favor of teachers with a health professional.

For the Commitments dimension, in the Attitudes section, 62.3% of positive responses were obtained among teachers who had a school nurse at their center, compared to 46.6% among those who did not have one (*p* = 0.036), for an adjusted OR of positive response of 1.89 (95% CI: 1.04–3.46; *p* = 0.038) in favor of teachers with a health professional.

For the Obligations dimension, in the Attitudes section, 77.3% of positive responses were obtained among the teachers who had this health professional in their center, compared to 64.5% among those who did not (*p* = 0.043), for an adjusted OR positive response of 1.87 (95% CI: 1.01–3.44; *p* = 0.045) in favor of teachers with a health professional.

For the Professional Burnout dimension, belonging to the Work Feelings section, 57.1% of positive responses were obtained among teachers who did not have a health professional at the center, compared to 45.7% among those who did (*p* = 0.040), for an adjusted OR of positive response of 0.63 (95% CI: 0.41–0.98; *p* = 0.041) in favor of teachers with a health professional (See Appendix A, Table A5).

For the rest of the dimensions and overall scores of the three sections, the differences in the answers given between the teaching staff with and without a nurse at the educational center did not reach statistical significance at the predetermined level.

## 4. Discussion

In the contingency plan model of the Ministry of Education, the figure of the school nurse for case management or to adapt the centers to the hygienic-sanitary measures established in the protocol itself is not contemplated [6]. However, a “COVID responsible or reference” was established (a designated teacher in each center, more than likely without health or risk prevention training) who acted as an interlocutor with the health services and who had to know the communication mechanisms established with the responsible health services in their territorial area. Thus, it is likely that the hygienic-sanitary and safety measures were taken by the teachers themselves in the majority of educational centers in the Canary Islands, as well as the handling and management of possible cases of infection by COVID-19, without any supervision of health professionals [6].

Prior to the outbreak of the pandemic, it had been shown that nurses in educational centers reduced school absenteeism rates by monitoring chronic illnesses in students and preventing infections [20]. Thus, now, in the midst of a pandemic, this professional would play a relevant role in planning health and safety protocols in schools, making decisions based on evidence to protect the safety of teachers and students [21,22]. In Spain, the nurses who carry out their interventions in the field of education are not legislated by any state law that regulates their presence in educational centers, nor to which body they depend (Health, Education, etc.) [10].

After an academic year with face-to-face activity, confidence among teachers about the availability of PPE has increased, as slightly more than half of those surveyed were considered as having the necessary PPE, while, in the previous study, at the beginning of the 2020/21 academic year, there were almost four out of five teachers who did not consider that PPE would be available in their workplace [4]. With the obtained results, we could state that, during the fifth wave of COVID-19 in the Canary Islands, the teachers who had this health professional in their workplace had the perception of being safer, as they were 1.81 times more positive in their responses in terms of the Coping Capacity dimension in their work environment, which denotes a greater perception of having the means to avoid contagion.

In addition, the presence of this health professional improved the perception that the salary they received for their work was more in line with the functions performed, and they believed that the educational system had coped better with the pandemic compared to the group of teachers who did not have a health professional in their centers. This is probably because the teacher who has this professional in their workplace has greater confidence that the infection prevention policies were based on practical evidence, as stated by Maughan et al. [23].

Regarding Attitudes, teachers who worked with a school nurse during the pandemic had a much better perception (up to three times higher) in the Risk dimension; in addition to perceiving more positively (almost twice as often) the dimensions of Commitments and Obligations towards their work. Based on these results, it could be said that these teachers are more willing to continue teaching despite the possibility of becoming infected, are more committed to their work, and are more willing to fulfill their obligations. For example, this group of teachers considered, in a higher proportion, that they had a duty to continue working despite the risk of becoming infected or infecting their family members, unlike their colleagues who did not have this health professional; these latest results are congruent to those obtained in the 2020 study [4].

In addition, having this healthcare professional at their center leads them to perceive to a greater extent (up to double) that their company has provided them with the necessary means to continue performing their duties as a teacher. This better perception in the analyzed dimensions could be related to the possibility that the nurse “released” the teachers from the additional workload in terms of infection prevention or management of possible cases, which were not part of their teaching skills [24]. In fact, Baisch et al. emphasized in their study the amount of time that a school nurse present at the school saves teachers on issues related to healthcare dedicated to students [25].

Regarding the Work Feelings dimension, we found that, in general, more than half of the teachers responded positively to the items that make up the Professional Burnout dimension. Similar results were reported by Kotowski et al. with a sample of 703 teachers from Cincinnati (USA), who showed how the pandemic had negatively influenced their work, as they spent up to 60% more time preparing classes and their mental health had worsened, thus increasing their levels of stress and burnout since the health crisis began [26]. However, the teachers who had this health professional at their center had (statistically) significantly less feelings of professional burnout, so having a health professional at the educational center became a protective factor to reduce work stress among school teachers.

Various studies conclude that administrations should support teachers, improving their working conditions, as these positively or negatively influence the physical, mental, and emotional well-being of workers [27,28,29]. Therefore, the public health and educational administrations of Spain should take into account the results of the present study in order to assess the homogeneous implementation and progressive regulation of the figure of the school nurse in the classroom, in order to reduce the extra workload for teachers (from non-teaching activities) that has arisen from the pandemic, without forgetting the role it can play in the health education within the educational community [12].

### 4.1. Future Proposals

The most relevant finding of our research is, without a doubt, that teachers who have a health professional in their schools, at least during a health crisis, have a greater commitment to their work and show less professional burnout. We believe that the progressive implementation of the health professional in educational centers could be a cornerstone to improve our educational system and the health status of students and teachers. This measure could increase student learning and improve the school environment, as authors such as Federici et al. have already shown [30,31].

The need to introduce the health professional in our educational system is proven as Spain has the second highest rate of early school absenteeism in the European Union and is also the second European country with the highest rate of childhood and youth obesity in Europe [32,33]. Thus, according to Best et al.’s study, the presence of a school nurse in educational centers (with adequate ratios) improves academic results and healthy habits of students, and reduces school absenteeism [34].

### 4.2. Strengths and Limitations

As a strength, it should be noted that this is the first study carried out in Spain that investigates the influence of the presence of a professional with health training among the teaching staff during a pandemic. Initially, a sample size of 469 participants was calculated, finally 640 participants took part in the study, which indicates the involvement of teachers in issues related to health in general and the pandemic in particular.

There are no official data that indicate exactly how many educational centers have a professional with health training. For this reason, we cannot verify whether we have reached the minimum sample size for the results to be generalizable, as only 94 teachers indicated that they did have the figure of school nurse in their educational center compared to 546 who indicated that they did not have this professional; it is evident that this represents a limitation to the study. However, this factor is relevant, as it indicates the low number of teachers who had a health professional in their centers that could control the possible cases among teachers and students in the second face-to-face academic year of the pandemic.

The results presented here show a glimpse of how the Canarian teaching staff was, mentally, during the end of the fifth wave and in the first months of the third academic year in the pandemic, when the accumulated incidence was declining and more than 85% of the general population had been vaccinated [35].

## 5. Conclusions

The main conclusion of the present study is that the teachers who had this health professional in their educational centers felt more protected, had a greater commitment to their work, and had less feelings of professional burnout. Therefore, having a health professional has influenced the educational field, promoting a positive coping with the difficulties experienced during the pandemic.

These results indicate that having nurses in the educational setting has a protective effect against some of the negative effects explored in this study. In addition, there is an improvement in coping and in the work commitment of teachers. These data should be taken into account by the health and educational administrations in order to assess the homogeneous and regulated implementation of the figure of the school nurse in the classrooms, being able to reduce for teachers the non-teaching workload derived from the pandemic, without forgetting the role that the presence of this health professional can play in relation to health education for the educational community.

These effects can be maintained after the pandemic, evolving towards other educational-health needs that both students and teachers need in the future. This must be contrasted with new research that takes into account new realities in relation to the positive contribution when it comes to teaching and developing healthy lifestyles.

## Data Availability

The data presented in this study are available on request from the corresponding author. The data are not publicly available because they belong to a doctoral thesis not yet defended.

## Appendix A

**Table A1:** Items distributed by dimensions and sections of the questionnaire.

Beliefs		
Dimensions	Coping Capacity	Do you think that the structure of the Spanish education system should be changed after this pandemic?
		Do you think that the COVID-19 pandemic has highlighted the shortcomings of the education system?
		Do you think that the education system has dealt with the pandemic adequately?
		Do you consider that the salary you receive is adjusted to the functions you perform at work?
		Do you currently have the appropriate PPE to protect you from contracting this pandemic disease?
	Prevention	Do you think current vaccines will help end the COVID-19 pandemic?
		Do you think that all teaching staff should be vaccinated against COVID-19?
	Changes Organization	Did you have to teach by telematic means during the 2020/21 academic year?
		Have your hours changed due to the pandemic?
Attitudes		
Dimensions	Risks	If there was a higher than usual risk of becoming infected at work and contracting COVID-19.
		If there was a higher than usual risk of infecting your family.
		If a colleague from your work team had died from COVID-19.
		If due to work needs, you lacked family-work conciliation.
		If your co-workers had been infected with COVID-19.
		If there was no possibility of maintaining the physical safety distance.
		If a family member had died from COVID-19.
		If you were proposed/asked to teach in groups with normal ratios (not COVID-19).
		If I had to work with the appropriate hygienic measures (minimum interpersonal distance, reduced student ratio, hydroalcoholic solution).
		Responsibility at work is above your family duties.
	Commitments	If there was no possibility of having a hydroalcoholic solution inside the classroom or in the common areas.
		If you were asked to work more hours.
		If you had to work with the appropriate protection measures (hygienic mask, surgical mask, protection screens, etc.).
		If you were asked to teach groups in which the students require greater closeness (students with SEN/SESN, infants).
		People who have worked during this time of health crisis must be rewarded in some way.
		Working during this time of pandemic has been the most important challenge I have faced during my working life.
		The implementation of a reference health professional in the workplace would have helped to better control possible outbreaks of COVID-19 among teachers and students.
	Obligations	If you received in return some kind of incentive from the company.
		All teaching staff have a duty to work during the pandemic even if there is a greater risk to their health.
		My company has provided me with the necessary material means to carry out my teaching function.
		People who refuse to work during this time of health crisis must be punished in some way.
		Teaching staff should have priority over the general population to be diagnosed and receive treatment during the pandemic.
		Vaccination against COVID-19 should be mandatory for teaching staff.
Feelings toward work		
Dimensions	Professional Burnout	Because of my job I feel emotionally exhausted.
		I feel physically and psychologically exhausted when leaving work.
		At work I feel that I am at the limit of my possibilities.
		I feel “burned out” by my current job.
		I have had a hard time sleeping at night since the pandemic started.
		I have been afraid of catching it while working.
		I lack time to finish all my tasks during the workday.
		My biggest fear is infecting my loved ones.
	Support	My institution adequately values my work as a teacher.
		I feel supported by my most direct superiors.
		Society recognizes my work as a teacher.
		I have possibilities of professional advancement in my current position.
	Work Satisfaction	Through my work, I feel that I am positively influencing other people’s lives.
		I consider that my work is useful for society.
		Despite everything, the pandemic has not eroded my vocation. If I went back in time, I would choose my profession again.
		I am very satisfied with my job.

**Table A2:** Demographic and employment information of the participants in general and according to whether or not they have a healthcare professional in their center.

Characteristics		Teachers with a School Nurse	Teachers without a School Nurse	*p*-Value *
Gender	Women	66 (70.2%)	411 (75.4%)	0.317
	Men	28 (29.8%)	130 (23.8%)	
	Not identified	-	5 (0.9%)	
Median Age (P_25_–P_75_)		51 (43–56)	49 (41–55)	0.018
Type of Education Center **	Infant-Primary-Special Education	33 (35.1%)	253 (46.3%)	0.007
	Secondary	39 (41.5%)	228 (41.8%)	
	Prof. Degrees-Univ-Other	22 (23.4%)	65 (11.9%)	
Educational Sector	Public	90 (95.7%)	520 (95.2%)	0.369
	Private	2 (2.1%)	9 (1.6%)	
	Subsidized	1 (1.1%)	16 (2.9%)	
	More than one type	1 (1.1%)	1 (0.2%)	
Type of Contract	Temporary or Substitute	5 (5.3%)	39 (7.1%)	0.311
	Interim	19 (20.2%)	144 (26.4%)	
	Public Servant	70 (74.5%)	363 (66.5%)	
Family Situation	I live with my partner and children	50 (53.2%)	243 (44.5%)	0.084
	I live with my partner	19 (20.2%)	113 (20.7%)	
	I live alone	8 (8.5%)	72 (13.2%)	
	I live alone and with children	14 (14.9%)	56 (10.3%)	
	I live with my parents or other relatives	2 (2.1%)	37 (6.8%)	
	I live with friends	1 (1.1%)	0 (0.0%)	
	I live in a family group (more than one answer)	0 (0.0%)	25 (4.6%)	
Time Worked in Education	<5 years	10 (10.6%)	83 (15.2%)	0.109
	6–10 years	9 (9.6%)	63 (11.6%)	
	11–15 years	13 (13.8%)	71 (13.0%)	
	16–20 years	14 (14.9%)	85 (15.6%)	
	21–25 years	14 (14.9%)	123 (22.6%)	
	26–30 years	16 (17.0%)	58 (10.6%)	
	>30 years	18 (19.1%)	74 (11.6%)	

* Comparison made with Pearson’s chi-squared test, Fisher’s exact test, or Mann–Whitney U test, as appropriate. ** Teachers’ responses are grouped as follows: (1) Infant-Primary-Special Ed. (Crèche 0–3 years + Preschool and Primary school + Special education center); (2) Secondary; (3) Prof. Degree-UNI-Other (Professional education center + University + Other: Official language school, adult centers, specific special education centers, etc.).

**Table A3:** Information regarding the COVID-19 pandemic on teachers in general and according to whether or not they have a healthcare professional in their center.

Information Related to the COVID-19 Pandemic	Teachers with School Nurse	Teachers without School Nurse	*p*-Value *
Do you live with vulnerable people or people with pathologies considered to be at risk (hypertension, diabetes, obesity, immunosuppressive treatment, COPD) of suffering from COVID-19 infection? (YES)	26 (27.7%)	218 (39.9%)	0.024
Are you a vulnerable person or with pathologies considered at risk (hypertension, diabetes, obesity, immunosuppressive treatment, COPD) against COVID-19 infection? (YES)	14 (14.9%)	148 (27.1%)	0.012
Have you been vaccinated against COVID-19? (YES)	91 (96.8%)	531 (97.3%)	0.738
Did you get COVID-19? (YES)	8 (8.5%)	20 (3.7%)	0.051
Did you have to undergo a home isolation process at some point during the pandemic (either due to contact with positive people or due to compatible symptoms)? (YES)	30 (31.9%)	160 (29.3%)	0.609
Have you required psychological or psychiatric support at any time during the pandemic? (YES)	12 (12.8%)	59 (10.8%)	0.576

* Comparison made with Pearson’s chi-squared test, Fisher’s exact test, or Mann–Whitney U test, as appropriate.

**Table A4:** Answers given to the items in the sections of the questionnaire globally and according to whether or not the teachers have a health professional in their educational center (only those with a significant difference are shown).

Item		Global Answers	Teachers with School Nurse	Teachers without School Nurse	*p*- Value ^1^	OR (IC95%) ^2^	*p*- Value ^3^
Beliefs	Do you currently have the appropriate personal protective equipment (PPE) to protect you from contracting this pandemic disease? (YES)	347 (58.0%)	63 (71.6%)	284 (55.7%)	0.005	2.03 (1.23–3.35)	0.006
	Do you think that the education system has dealt with the pandemic adequately? (YES)	320 (56.0%)	58 (66.7%)	262 (54.1%)	0.030	2.00 (1.28–3.12)	0.030
	Do you consider that the salary you receive is adjusted to the functions you perform at work? (YES)	271 (44.1%)	54 (58.1%)	217 (41.6%)	0.003	1.95 (1.24–3.04)	0.004
Attitudes	If there was a higher than usual risk of becoming infected at work and contracting COVID-19. (PROBABLE)	138 (28.7%)	28 (38.4%)	110 (27.0%)	0.049	1.68 (1.01–2.83)	0.049
	If there was a higher than usual risk of infecting your family. (PROBABLE)	84 (17.1%)	20 (27.8%)	64 (15.3%)	0.009	2.12 (1.19–3.81)	0.010
	All teaching staff have a duty to work during the pandemic even if there is a greater risk to their health. (AGREE)	209 (32.7%)	41 (43.6%)	168 (30.8)	0.014	1.74 (1.11–2.72)	0.015
	People who refuse to work during this time of health crisis must be punished in some way. (AGREE)	202 (31.6%)	41 (43.6%)	161 (29.5%)	0.006	1.85 (1.18–2.89)	0.007
	My company has provided me with the necessary material means to carry out my teaching function. (AGREE)	360 (56.3%)	64 (68.1%)	296 (54.2%)	0.012	1.80 (1.13–2.87)	0.013

^1^ Comparison made with Pearson’s chi-squared test, Fisher’s exact test, or Mann–Whitney U test, as appropriate. ^2^ Estimated with multivariable binary logistic regression adjusted for sociodemographic and employment factors with statistical significance *p* ≤ 0.10 between responses. ^3^ Backward stepwise estimation from the full model and the Wald adjustment strategy.

**Table A5:** Answers given to the dimentions of the questionnaire globally and according to whether or not the teachers have a health professional in their educational center.

Dimensions		Global Answers	Teachers with School Nurse	Teachers without School Nurse	*p*- Value ^1^	OR (IC95%) ^2^	*p*- Value ^3^
Beliefs	Coping Capacity	146 (31.1%)	29 (42.6%)	117 (29.2%)	0.027	1.81 (1.07–3.06)	0.028
	Prevention	425 (94.7%)	69 (93.2%)	356 (94.9%)	0.571	---	---
	Changes Organization	330 (52.2%)	53 (56.4%)	277 (51.5%)	0.381	---	---
	Global Beliefs	151 (44.4%)	31 (59.6%)	120 (41.7%)	0.017	2.07 (1.13–3.77)	0.018
Attitudes	Risks	27 (18.9%)	10 (33.3%)	17 (15.0%)	0.023	2.82 (1.13–7.07)	0.027
	Commitments	162 (49.1%)	33 (62.3%)	129 (46.6%)	0.036	1.89 (1.04–3.46)	0.038
	Obligations	304 (66.4%)	51 (77.3%)	253 (64.5%)	0.043	1.87 (1.01–3.44)	0.045
	Global Attitudes	54 (57.4%)	17 (77.3%)	37 (51.4%)	0.032	3.21 (1.07–9.65)	0.037
Feelings toward work	Professional Burnout	355 (55.5%)	43 (45.7%)	312 (57.1%)	0.040	0.63 (0.41–0.98)	0.041
	Support	315 (41.9%)	53 (56.4%)	262 (48.0%)	0.133	---	*---*
	Work Satisfaction	568 (88.8%)	85 (90.4%)	483 (88.5%)	0.578	---	*---*
	Global Work Feelings	586 (91.6%)	83 (88.3%)	503 (92.1%)	0.218	---	*---*

^1^ Comparison made with Pearson’s chi-squared test, Fisher’s exact test, or Mann–Whitney U test, as appropriate. ^2^ Estimated with multivariable binary logistic regression adjusted for sociodemographic and employment factors with statistical significance *p* ≤ 0.10 between responses. ^3^ Backward stepwise estimation from the full model and the Wald adjustment strategy.

## References

[1] Real Decreto 463/2020, de 14 de Marzo, Por el Que se Declara el Estado de Alarma Para la Gestión de la Situación de Crisis Sanitaria Ocasionada Por el COVID-19. Boletín Oficial del Estado, Número 67, (14 de marzo de 2020). https://www.boe.es/buscar/pdf/2020/BOE-A-2020-3692-consolidado.pdf.

[2] García-Peñalvo F.J., Corell A., Abella-García V., Grande M. (2020). La evaluación online en la educación superior en tiempos de la COVID-19. Educ. Knowl. Soc..

[3] Solís García P., Lago Urbano R., Real-Castelao S. (2021). Consequences of COVID-19 Confinement for Teachers: Family-Work Interactions, Technostress, and Perceived Organizational Support. Int. J. Environ. Res. Public Health.

[4] Santana-López B.N., Santana-Padilla Y.G., Santana-Cabrera E.G., Ruiz-Rodríguez G.R., González-Martín J.M., Santana-Cabrera L. (2021). Actitudes y conocimientos sobre la pandemia por la COVID-19 en docentes de Canarias. Rev. Peru. Med. Exp. Salud Publica.

[5] Santana-Cabrera L., Santana-Cabrera E.G., Santana-López B.N. (2020). Medidas a implantar en la vuelta a la escuela en período COVID-19. Rev. Esp. Salud Pública..

[6] Consejería de Educación, Universidades, Cultura y Deportes del Gobierno de Canarias Modelo de Plan de Contingencia Frente a la COVID-19 en los Centros Educativos no Universitarios de Canarias, Curso Académico 2020–21. https://www.gobiernodecanarias.org/cmsweb/export/sites/educacion/web/_galerias/descargas/covid/2020-09-01__Modelo-Plan-de-Contingencia-COVID_1.pdf.

[7] Pressley T., Ha C., Learn E. (2021). Teacher stress and anxiety during COVID-19: An empirical study. Sch. Psychol..

[8] Ozamiz-Etxebarria N., Berasategi-Santxo N., Idoiaga-Mondragon N., Dosil-Santamaría M. (2021). The Psychological State of Teachers During the COVID-19 Crisis: The Challenge of Returning to Face-to-Face Teaching. Front. Psychol..

[9] Almendros-Irala A. (2022). España Suspende en Enfermería Escolar: Una Enfermera Por Cada 8.500 Alumnos. [Internet]. España: Consejo General de Enfermería—Departamento de Comunicación. https://www.consejogeneralenfermeria.org/actualidad-y-prensa/sala-de-prensa/notas-prensa/send/20-notas-de-prensa/1647-espana-suspende-en-enfermeria-escolar-una-enfermera-por-cada-8-500-alumnos.

[10] Asociación Catalana de Enfermería y Salud Escolar, Asociación Científica Española de Enfermería y Salud Escolar (2022). Marco contextual de la enfermería escolar en el ámbito internacional y nacional. Girona. https://www.adenyd.es/wp-content/uploads/2022/06/5b834533-ded1-43f9-9ddc-d7d71d453d12_compressed.pdf.

[11] Durant B.V., Gibbons L.J., Poole C., Suessmanm M., Wyckoff L. (2011). NASN position statement: Caseload assignments. NASN Sch. Nurse..

[12] Best N.C., Oppewal S., Travers D. (2018). Exploring School Nurse Interventions and Health and Education Outcomes: An Integrative Review. J. Sch. Nurs..

[13] Martinsson E., Garmy P., Einberg E.-L. (2021). School Nurses’ Experience of Working in School Health Service during the COVID-19 Pandemic in Sweden. Int. J. Environ. Res. Public Health.

[14] Martinsson E., Garmy P., Einberg E.-L. (2022). School Nurses’ Perceptions About Student’s Wellbeing During the COVID-19 Pandemic in Sweden. J. Sch. Nurs..

[15] Maughan E.D. (2016). Building Strong Children: Why We Need Nurses in Schools. Am. Educ..

[16] Instituto de Salud Carlos III Informe nº 98: Situación de COVID-19 en España a 29 de Septiembre de 2021. Madrid..

[17] Instituto de Salud Carlos III Informe nº 102: Situación de COVID-19 en España a 27 de Octubre de 2021. Madrid..

[18] ISTAC [Internet] Canarias: Instituto Canario de Estadística; 2022. Tablas de Profesorado no Universitario Según Titularidad de Los Centros, Dedicación o Enseñanzas de la Provincias de Canarias [Dedicación o Enseñanzas: Total, Provincias: Canarias, Titularidad: Total]. http://www.gobiernodecanarias.org/istac/jaxi-istac/tabla.do?uripx=urn:uuid:f25d4cc7-5190-4276-93e4-af1c08737b0a.

[19] ISTAC [Internet] Canarias: Instituto Canario de Estadística; 2022. Tablas de Personal Docente e Investigador de Los Centros Propios Según Universidades Públicas, Sexos, Categorías y Cursos. Canarias. [Universidades: Total, Sexos: Ambos sexos, Categorías: Total, Cursos: 2019/20]. http://www.gobiernodecanarias.org/istac/jaxi-istac/tabla.do?uripx=urn:uuid:289b50a1-a504-4fdf-9c44-6416e034540f&uripub=urn:uuid:b9fb42be-727a-4a35-a24a-002ce7b6612c.

[20] Yoder C.M. (2020). School Nurses and Student Academic Outcomes: An Integrative Review. J. Sch. Nurs..

[21] Hoke A.M., Keller C.M., Calo W.A., Sekhar D.L., Lehman E.B., Kraschnewski J.L. (2021). School Nurse Perspectives on COVID-19. J. Sch. Nurs..

[22] The Lancet (2021). COVID-19: The intersection of education and health. Lancet..

[23] Maughan E.D., Johnson K.H., Gryfinski J., Lamparelli W., Chatham S., Lopez-Carrasco J. (2021). Show Me the Evidence: COVID-19 and School Nursing in the 21st Century. NASN Sch. Nurse.

[24] Shin E.M., Roh Y.S. (2020). A School Nurse Competency Framework for Continuing Education. Healthcare.

[25] Baisch M.J., Lundeen S.P., Murphy M.K. (2011). Evidence-based research on the value of school nurses in an urban school system. J. Sch. Health.

[26] Kotowski S.E., Davis K.G., Barratt C.L. (2022). Teachers feeling the burden of COVID-19: Impact on well-being, stress, and burnout. Work.

[27] Palma-Vasquez C., Carrasco D., Hernando-Rodriguez J.C. (2021). Mental Health of Teachers Who Have Teleworked Due to COVID-19. Eur. J. Investig. Health Psychol. Educ..

[28] Giorgi G., Lecca L.I., Leon-Perez J.M., Pignata S., Topa G., Mucci N. (2020). Emerging Issues in Occupational Disease: Mental Health in the Aging Working Population and Cognitive Impairment—A Narrative Review. BioMed Res. Int..

[29] Silva N.S.S.E., Elizabeth-Cabral B.R., Leão L.L., Pena G.D.G., Pinho L., Magalhães T.A. (2021). Working conditions, lifestyle and mental health of Brazilian public-school teachers during the COVID-19 pandemic. Psychiatriki.

[30] Federici R.A., Flatø M., Bru L.A., Midthassel U.V., Helleve A., Rønsen E. (2019). Can school nurses improve the school environment in Norwegian primary schools? A protocol for a randomized controlled trial. Int. J. Educ. Res..

[31] Helleve A., Midthassel U.V., Federici R.A. (2022). Finding the Balance Between Collaboration and Autonomy Among School Nurses in Interactions With Schools. J. Sch. Nurs..

[32] Instituto Nacional de Estadística [Internet] Abandono Temprano de la Educación-Formación. https://www.ine.es/ss/Satellite?L=es_ES&c=INESeccion_C&cid=1259925480602&p=1254+35110672&pagename=ProductosYServicios%2FPYSLayout.

[33] López-Sobaler A.M., Aparicio A., Salas-González M.D., Loria-Kohen V., Bermejo-López L.M. (2021). Obesidad en la población infantil en España y factores asociados. Nutr. Hosp..

[34] Best N.C. (2021). Nichols, A.O.; Waller, A.E., Zomorodi, M.; Pierre-Louis, B.; Oppewal. S. Impact of School Nurse Ratios and Health Services on Selected Student Health and Education Outcomes: North Carolina, 2011–-2016. J. School Health.

[35] Ministerio de Sanidad (2021). Informe de Actividad del Proceso de Vacunación: 25 de Octubre de 2021. https://www.sanidad.gob.es/profesionales/saludPublica/ccayes/alertasActual/nCov/documentos/Informe_GIV_comunicacion_20211025.pdf.

